# Evaluation of outbreak persistence caused by multidrug-resistant and echinocandin-resistant *Candida parapsilosis* using multidimensional experimental and epidemiological approaches

**DOI:** 10.1080/22221751.2024.2322655

**Published:** 2024-02-21

**Authors:** Farnaz Daneshnia, Daniel J. Floyd, Adam P. Ryan, Pegah Mosharaf Ghahfarokhy, Arefeh Ebadati, Sebastian Jusuf, Julieta Munoz, Nathan Elias Jeffries, Emma Elizabeth Yvanovich, Anna Apostolopoulou, Austin M. Perry, Cornelia Lass-Flörl, Asuman Birinci, Süleyha Hilmioğlu-Polat, Macit Ilkit, Geraldine Butler, Clarissa J. Nobile, Amir Arastehfar, Michael K. Mansour

**Affiliations:** a Division of Infectious Diseases, Massachusetts General Hospital, Harvard Medical School, Boston, MA, USA; b Institute of Biodiversity and Ecosystem Dynamics (IBED), University of Amsterdam, Amsterdam, The Netherlands; c School of Biomolecular and Biomedical Science, Conway Institute, University College Dublin, Belfield, Dublin, Ireland; d Department of Molecular and Cell Biology, School of Natural Sciences, University of California – Merced, Merced, CA, USA; e Health Sciences Research Institute, University of California – Merced, Merced, CA, USA; f Department of Medicine, Harvard Medical School, Boston, MA, USA; g Center for Regenerative Medicine, Boston, Massachusetts, USA; h Medical University Innsbruck, Institute of Hygiene and Medical Microbiology, Innsbruck, Austria; i Department of Medical Microbiology, Faculty of Medicine, Ondokuz Mayıs University, Samsun, Türkiye; j Department of Medical Microbiology, Ege University Faculty of Medicine, Izmir, Türkiye; k Division of Mycology, Faculty of Medicine, Çukurova University, Adana, Türkiye

**Keywords:** Multidrug resistance, echinocandin resistance, mannan, chitin, Β-glucan

## Abstract

*Candida parapsilosis* is known to cause severe and persistent outbreaks in clinical settings. Patients infected with multidrug-resistant *C. parapsilosis* (MDR Cp) isolates were identified in a large Turkish hospital from 2017–2020. We subsequently identified three additional patients infected with MDR Cp isolates in 2022 from the same hospital and two echinocandin-resistant (ECR) isolates from a single patient in another hospital. The increasing number of MDR and ECR isolates contradicts the general principle that the severe fitness cost associated with these phenotypes could prevent their dominance in clinical settings. Here, we employed a multidimensional approach to systematically assess the fitness costs of MDR and ECR *C. parapsilosis* isolates. Whole-genome sequencing revealed a novel MDR genotype infecting two patients in 2022. Despite severe *in vitro* defects, the levels and tolerances of the biofilms of our ECR and MDR isolates were generally comparable to those of susceptible wild-type isolates. Surprisingly, the MDR and ECR isolates showed major alterations in their cell wall components, and some of the MDR isolates consistently displayed increased tolerance to the fungicidal activities of primary human neutrophils and were more immunoevasive during exposure to primary human macrophages. Our systemic infection mouse model showed that MDR and ECR *C. parapsilosis* isolates had comparable fungal burden in most organs relative to susceptible isolates. Overall, we observed a notable increase in the genotypic diversity and frequency of MDR isolates and identified MDR and ECR isolates potentially capable of causing persistent outbreaks in the future.

## Introduction

*Candida parapsilosis* is one of the major causes of candidemia, ranking as the second to third most common among *Candida* species according to country and patient age. Although once considered a trivial clinical challenge, studies emerging from 2010 onward showed an increasing rate of antifungal drug resistance in *C. parapsilosis* isolates. Currently, outbreaks due to fluconazole-resistant (FLCR) *C. parapsilosis* isolates are recognized as a clinical challenge in multiple European, Asian, African, and South American countries [1]. Similarly, new studies conducted in healthcare settings in the United States [2] and Canada [3] detected an increasing number of patients infected with FLCR isolates, whereas the incidence of candidemia due to *C. parapsilosis* has remained largely stable [2]. Patients infected with resistant isolates have a poor prognosis [4], and the FLCR isolates cause persistent outbreaks despite the use of extensive environmental decontamination procedures [5]. These studies indicate that the emergence of drug resistance needs to be taken seriously early on, with the hope that prompt and appropriate clinical measures could hinder and/or decelerate the spread of drug-resistant pathogens.

The clinical importance of *C. parapsilosis* goes beyond FLCR challenges. For example, the emergence of echinocandin-resistant (ECR) *C. parapsilosis* isolates in China [6] and Greece [7] has undermined the efficacy of echinocandins, the frontline antifungal drugs. The emergence of small outbreaks caused by multidrug-resistant (MDR) *C. parapsilosis* isolates (resistant to ≥2 agents belonging to different classes of antifungal drugs) in Türkiye is even further concerning [8,9]. Indeed, through the application of whole-genome sequencing (WGS), we have noted that a series of pediatric patients have been infected with MDR strains of the same genotype [8,9], indicating that this seemingly small and emerging outbreak could be a matter of serious concern in the future. Patients infected with these MDR strains cannot be treated with fluconazole and echinocandins, and the sole antifungal drug able to clear these systemic infections is amphotericin B (AMB) [8,9]. These studies underscore how drug resistance has the potential to complicate clinical outcomes and render the two most widely used antifungal drug classes useless.

FLCR is mostly due to the acquisition of mutations in the drug target Erg11, with *C. parapsilosis* isolates harbouring Y132F and Y132F + K143R as the most common mutations [1]. Our team and others have found that the acquisition of certain mutations in the transcription factors controlling efflux pumps, such as Cdr1 and Mdr1, can also drive FLCR in *C. parapsilosis* [10,11]. ECR in *C. parapsilosis* isolates is known to occur through the acquisition of mutations in a short stretch of the drug target catalytic units of the Fks1 β-glucan synthase, known as hotspot 1 (HS1) and hotspot 2 (HS2) [1]. Given that these enzymes play important roles in regulating overall cellular function and homeostasis, it is plausible that drug-resistant *C. parapsilosis* isolates could be outcompeted by their susceptible counterparts, which would subsequently decrease their prevalence in clinical settings. Maintaining this assumption, several studies have shown that ECR isolates of *Candida albicans* carry dramatic fitness costs, such as reduced growth rates and filamentation abilities. Therefore, these isolates can be easily outcompeted by their susceptible counterparts during systemic infections [12]. Accordingly, the rare prevalence of ECR in *C. albicans* in clinical settings is attributed to the severe fitness costs accompanying drug resistance [12]. Similarly, *in vitro* generated ECR *C. parapsilosis* isolates carrying various mutations in Fks1 have been found to be significantly less virulent than susceptible wild-type isolates [13]. These findings beg the question, why has there been an incremental increase in the number of MDR *C. parapsilosis* infections, which should carry a higher fitness cost than ECR isolates?

Here, we continued to analyze *C. parapsilosis* infections at Ege University Hospital (EUH) in Türkiye, the same hospital in which the first detection of drug resistant *C. parapsilosis* outbreaks occurred [8,9]. Three additional patients infected with MDR isolates were next identified. A series of susceptible and ECR isolates from the same patient were characterized, in which the initial susceptible isolate was replaced by an ECR isolate during the infection, and before echinocandin treatment. We speculated that the persistence of the MDR and ECR strains could be due to either greater biofilm formation abilities, which allows persistence within hospital or possibly host environments, or better adaptation to host conditions, allowing them to persistently cause infections. WGS analysis revealed the persistence of the previously identified MDR genotype and the emergence of a novel MDR genotype, potentially descending from the same parent. Based on extensive *in vitro*, *ex vivo*, and *in vivo* analyses, our data suggests that ECR isolates and some MDR isolates do not suffer from obvious fitness costs, especially in the context of *in vivo* systemic infection and biofilm formation. Combined with our epidemiological data, this study provides evidence for the persistence of ECR and MDR *C. parapsilosis* isolates in the clinic. Accordingly, the utilization of such data could aid in devising and applying preventive strategies to decrease the spread and/or prevalence of such challenging drug-resistant *C. parapsilosis* isolates.

## Material and methods

### Clinical isolates and clinical data

The *C. parapsilosis* isolates and their microbiological data are listed in Table 1. In total, we included 13 *C. parapsilosis* isolates; 10 were collected from EUH, including 8 MDR isolates (5 previously published and 3 new isolates) and two fully susceptible isolates; and 3 isolates from Ondokuz Mayıs University Hospital, comprising 1 fully susceptible isolate and two ECR isolates. The clinical data available for the new isolates included in the current study are discussed below. These isolates were part of an ongoing multicenter epidemiological study. These studies were conducted in accordance with local legislation and institutional requirements. The experimental protocols were approved by the Ethics Committee of the Ege University Faculty of Medicine (20-2 T/30) and Ondokuz Mayıs University, Faculty of Medicine (2023/429).

**Table 1.** The microbiological data of the multidrug- and echinocandin-resistant and susceptible isolates included in this study. Except for isolate #314 (recovered from central venous catheter) the rest of the isolates were obtained from blood samples.

Original name	New names	Group names	Isolation year	Minimum inhibitory concentration (µg/ml)					Erg11 genotype	Fks1 genotype	Comments
				FLC	MICA	ANI	CAS	AMB			
Cp142	MDR0	MDR0	2017	64	>8	2	4	0.5	G458S	R658G	Used for in vivo
Cp196	MDR1	Historic MDR	2018	64	>8	2	4	0.5	Y132F + K143R	R658G	Used for in vivo
Cp120	MDR2	Historic MDR	2019	64	>8	2	4	0.5	Y132F + K143R	R658G	
Cp207	MDR3	Historic MDR	2019	64	>8	2	4	0.5	Y132F + K143R	R658G	Used for in vivo
110T	MDR4	Historic MDR	2020	8	>8	2	4	1	Y132F + K143R	R658G	
51	MDR5	Novel MDR	2021	64	>8	2	4	0.5	Y132F + G307A	W1370R	
93	MDR6	Historic MDR	2022	64	>8	2	4	1	Y132F + K143R	R658G	Used for in vivo
122	MDR7	Novel MDR	2022	64	>8	2	4	1	Y132F + G307A	W1370R	Used for in vivo
314	314	SX	2022	0.5	1	2	4	0.5	WT	WT	Used for in vivo
315	315	ECR	2022	1	8	2	4	0.5	WT	F652L	
316	316	ECR	2022	1	8	2	4	0.5	WT	F652L	Used for in vivo
1T	1T	S-Ege	2019	0.5	2	1	1	0.5	WT	WT	
26T	26T	S-Ege	2019	0.5	2	1	1	1	WT	WT	Used for in vivo

**Isolate MDR5:** A one-year-old male patient with past history of Hirschsprung’s disease was admitted to EUH on August 17, 2020 for management of his bowel complications.

The patient underwent three operations—bowel lengthening, jejunostomy, and ileostomy—during his hospitalization. Subsequently, he was hospitalized in the Pediatric Surgery Intensive Care Unit. During his hospitalization, bacteria (carbapenem-resistant *Klebsiella pneumoniae*, *Pseudomonas aeruginosa*, and *Staphylococcus epidermidis*) were recovered from his blood and urine cultures. He received broad-spectrum antibacterials, and prophylactic fluconazole. During a transfer to the pediatric intensive care unit, caspofungin was added empirically to the prophylactic fluconazole on June 21, 2021 (indication was not specified). *C. parapsilosis* was isolated from the patient’s urine and blood samples taken on June 26 and June 30, 2021, respectively, after which treatment with caspofungin was continued. Finally, the patient succumbed to infection on July 5, 2021.

**Isolate MDR6:** A 4.5-year-old male patient previously diagnosed with Shah-Waardenburg syndrome and Hirschsprung’s disease was transferred from another hospital to EUH on March 21, 2022 due to bilious vomiting and severe diarrhea admitted to EUH. The patient was febrile on March 22, 2022, and his urine and blood cultures were positive for *C. parapsilosis*. Treatment with fluconazole (80 mg daily for two days) was started on March 24, 2022, and then increased to 12 mg/kg on March 26, 2022 with the addition of liposomal amphotericin B; subsequently, the patient became afebrile on March 30, 2022. The patient had an indwelling venous catheter, which was removed on March 31, 2022, and fluconazole treatment was continued for another 14 days. The patient was discharged on April 13, 2022, and fluconazole treatment was continued for another 7 days. On the latest update (one month later), the patient returned to the hospital with persistent isolation of *C. parapsilosis* in multiple urine samples despite the long duration and high dosage of fluconazole treatment.

**Isolate MDR7:** A 14-month-old female patient was referred to the EUH with respiratory stress syndrome on September 28, 2022. Four months earlier she underwent complete correction of tetralogy of Fallot, atrial septal defect repair, and tracheostomy. Upon hospitalization at the EUH, tracheal aspirate swabs, bronchoalveolar lavage (BAL) fluid, and blood cultures were taken on September 28, 2022. The tracheal aspirate and BAL samples were positive for *Acinetobacter baumanii*, and a blood culture was positive for *C. parapsilosis*. Accordingly, gentamycin and caspofungin were started. Subsequent blood cultures taken on October 4, 2022, were again positive for *C. parapsilosis*; therefore, caspofungin was discontinued, and liposomal amphotericin B was added and continued until October 26, 2022. All the blood cultures taken during liposomal amphotericin B therapy were negative for *C. parapsilosis,* and the patient was discharged on November 1, 2022.

**Isolates 314-316:** A 59-year-old female patient was admitted to Ondokuz Mayıs University Hospital on February 6, 2022. Detailed past medical history data are lacking for this patient. She was immediately transferred to the neurosurgical service upon admission due to subarachnoid hemorrhage and aneurysm rupture. A central venous catheter was inserted on March 31, 2022. *C. parapsilosis* was confirmed via blood culture on April 14 (#314), and subsequent peripheral vein catheter (#315) and blood (#316) cultures were positive for *C. parapsilosis* on April 16, 2022. Caspofungin treatment was started on April 17 and continued until April 25, 2022. The patient was discharged on July 7, 2022; however, clinical and microbiological data relating to caspofungin treatment and discharge were missing.

### Antifungal susceptibility testing (AFST)

AFST was performed following the Clinical and Laboratory Standards Institute (CLSI) broth microdilution approach (CLSI-M27), which included fluconazole, micafungin, anidulafungin, and amphotericin B (AMB) (all sourced from Sigma, St. Louis, MO, USA). The 96-well plates containing the drug and the isolated suspensions were incubated for 24 hours at 37°C, after which the minimum inhibitory concentrations (MICs) were determined visually. Isolates with MIC values ≥8 µg/mL for fluconazole and echinocandins were denoted as fluconazole and echinocandin resistant, respectively. The MIC values for AMB were reported as wildtype (WT < 2 µg/mL) or non-WT (>2 µg/mL) given the lack of defined clinical breakpoints. *C. parapsilosis* (ATCC 22019) and *Pichia kudriavzevii* (ATCC 6258) type strains were included for quality control purposes.

### Whole-genome sequencing

Genomic DNA was isolated from *C. parapsilosis* strains MDR5-7, 314, 315 and 316 by phenol–chloroform extraction (Sigma-Aldrich P3803). Libraries were prepared using an Illumina DNA Prep Kit (Cat No. 20018704). DNA was sequenced using an Illumina NextSeq 2000 instrument in 150 bp paired-end format, producing ∼7.9-13.7 million reads per sample. Sequence information for all the other isolates was obtained as described in Supplementary Table 1 [9,14–17]. The raw reads for all the samples were trimmed to remove those with a mean quality <30 and minimum length <35 using Skewer (v.0.2.2) [18]. Reads were mapped to the *C. parapsilosis* CDC317 reference genome [15] using BWA (v. 0.7.12-r1039) [19]. BAM files were filtered to remove duplicates and sorted using Picard tools [20] and SAMtools (v.1.18) [21]. Variants were called in GVCF format using GATK (v. 4.0.1.2). HaplotypeCaller GVCF files for each sample were combined and genotyped using GATK CombineGVCFs and GATK GenotypeGVCFs [20]. Variants were filtered using GATK VariantFiltration [20] using the following parameters: minimum mapping qualities of 30, minimum genotype qualities of 40, minimum read depth of 30, and maximum read depth of 200. Only biallelic SNPs with no missing sites were retained. Copy number variants (CNVs) were determined from BAM alignments using Delly CNV (v. 0.8.7) [22]. The gene copy number was estimated from CNV segmentations using bedtools intersect (v. 2.30.0) [23]. For phylogenetic analysis, the filtered SNP VCF file was converted to FASTA format. The heterozygous sites were randomly assigned to either the reference or the alternative allele. All samples with a specific heterozygous variant were assigned the same allele. A phylogenetic tree was generated using RAxML (v. 8.2.12) [24] with the GTRGAMMA model of substitution and 1000 bootstrap replicates. The final tree was visualized using iTOL [25]. The same SNP matrix was utilized in a multiple correspondence analysis (MCA), which was conducted as described previously [9]. Variants were annotated using SIFT 4G [26] using a prediction database built for the *C. parapsilosis* reference CDC317 [15]. In total, 6985 protein-coding variants were identified across the sample set. This included 6771 amino acid changes, 9 start losses, 187 stop gains and 18 stop losses.

### *In vitro* growth rate measurement

Dynamic growth rates were determined using a previously described protocol [9]. Briefly, *C. parapsilosis* isolates grown overnight in Yeast Peptone Dextrose (YPD) broth (incubated in a 37°C incubator at 150 rpm) were washed thrice with 1x Phosphate Buffered Saline (PBS) and the optical density at 600 nm (OD_600_) was adjusted to 0.2 using the desired media, which included YPD (pH 7), YPD (pH 5), a broth medium containing glycerol instead of dextrose (YPG), YPD containing 10 mM H_2_O_2_, 0.02% sodium dodecyl sulfate (SDS), and 60 µg/mL Congo Red. 200 mL of the inoculum was transferred to 96-well plates, and the growth rate was monitored using a SpectraMax® i3x multimode detection platform (VWR) while the plates were incubated at 37°C. The growth rate data were analyzed with Microsoft Excel, and relevant graphs were generated using GraphPad Prism v7.00 software.

### Biofilm assays

Biofilm assays were performed according to previously described standard protocols, with slight modifications [27]. For the optical density (OD) biofilm assay, non-tissue culture treated sterile flat-bottomed 96-well polystyrene plates (Corning, Catalog #351143) were used to grow biofilms. The 96-well plates were incubated for 90 minutes at 37 °C with shaking at 250 rpm in an ELMI shaker (M2 Scientifics, Catalog #ELMI-TRMS04) after being seeded with *C. parapsilosis* cells at a final OD_600_ of 0.5 in a final volume of 200 μL Spider + 1% glucose medium. Following the first 90-minute adherence period, each well received a fresh dose of Spider + 1% glucose medium medium after a gentle wash with PBS. Breathable sealing membranes (Sigma Aldrich, Catalog #Z380059) were used to seal the plates prior to incubating at 37 °C with 250 rpm shaking in an ELMI shaker for 24 hours. Using a BioTek Epoch 2 plate reader (Agilent, Santa Clara, CA), the OD_600_ of each well was measured after the media was removed. The data were normalized by subtracting the OD_600_ value of average blank wells (containing media alone) from each experimental well and the means and standard deviations were calculated. The biofilm OD assay was performed using eight replicates per sample. For the confocal scanning laser microscopy (CSLM) biofilm assay, one autoclaved silicone square was placed per well into a 12-well plate using sterile forceps. Silicone squares were treated with fetal bovine serum (FBS) (Sigma Aldrich), for 24 hours at 200 rpm. Following cell density determination of overnight cultures of the *C. parapsilosis* strains, cells were added to the wells containing the silicone sequres at a final concentration of 1 × 10^7^ cells/mL in 2 mL Spider +1% glucose media in the 12-well plates. Plates were incubated at 37°C for 90 min at 200 rpm in an ELMI incubator. Media containing non-adhered cells were aspirated from each well and the wells were washed using 2 mL of sterile Dulbecco’s calcium and magnesium salt-free phosphate-buffered saline (DPBS). Fresh medium was added to each well and the plates were incubated at 37°C for 24 hours at 200 rpm. 80 μL of formaldehyde solution (36.5–38% in water) was added into each well for 20 minutes at 200 rpm in the ELMI incubator to fix the biofilms. Media was aspirated from each well and 2 mL of RPMI-1640 medium buffered with L-glutamine and 3-(N-morpholino) propane sulfonic acid (MOPS), without sodium bicarbonate, pH 7.0, was added to each well. 10 μL Concanavalin A (10 mg/mL; 50 μg/mL final concentration), Alexa Fluor 594 conjugate (Sigma Aldrich) was added into each well containing the fixed biofilms, and plates were shaken at 37°C for 60 minutes at 200 rpm. Strains were visualized using a 555 nm diode (red) laser. Z-Stacks were obtained at 652 × 652 pixels, imaging every 0.5 μm intervals using a water-dipping 40X objective lens. CSLM images in.czi format were obtained and analyzed using the project stacks function in ImageJ to construct top-views and side-views of each biofilm.

### Biofilm tolerance to hydrogen peroxide (H_2_O_2_, 100 mM) and sodium hypochlorite (bleach, 2.5%)

Biofilms were grown to assess tolerance to H_2_O_2_ and bleach as previously described [28]. Briefly, *C. parapsilosis* isolates grown overnight in YPD liquid medium were washed thrice with PBS, and their OD_600_ values were adjusted to 1.0 with fresh YPD, seeded in 48-well plates, and incubated for 24 hours at 37°C. Subsequently, the wells were washed with PBS to remove nonadherent cells, treated with H_2_O_2_ (100 mM) or bleach (2.5%), and incubated overnight (16-18 hrs) at 37°C. Duplicate untreated control samples were also included. Subsequently, the treated and untreated wells were washed carefully, PBS containing a nonlethal concentration of proteinase K (20 µg/mL) was added to each well, the biofilm structures were disrupted (the free yeast cells were clearly visible by light microscopy at a magnification of 40X), and the cell suspensions were plated onto YPD plates and incubated for 24 hours at 37°C. Colony forming units (CFUs) were counted 2 days after incubation on YPD plates, and survival was measured by dividing the CFU number of treated samples over the average of the untreated controls. The values are presented as percentages.

### Primary human monocyte isolation, differentiation into mature macrophages, and phagocytosis

Primary human monocytes were isolated from the leukopak of at least two healthy donors attending the Blood Component Processing Center in Massachusetts General Hospital following the approved IRB protocol (#2014P002377). After monolayer isolation using Ficoll (Thermo Fisher), the cells were subjected to the EasySep™ Human Monocyte Isolation Kit (STEMCELL Technologies). The purity and viability of the cells were further assessed by flow cytometry according to the manufacturer’s instructions. Fully differentiated macrophages from primary human monocytes were prepared using a previously described protocol with slight modifications [29]. Briefly, monocytes were treated with complete RPMI (cRPMI, 10% heat-inactivated FBS, 1% pen-strep, 1% L-glutamine, and 88% RPMI 1640) supplemented with 50 ng/mL human macrophage-colony stimulating factor (M-CSF) and incubated in a CO_2_ incubator at 37°C for 4 days. After this incubation, the differentiating macrophages were washed with warm PBS to detach the nonadherent/dead cells, treated with fresh cRPMI containing 50 ng/mL M-CSF, and incubated for another 72 hours in a CO_2_ incubator at 37°C. The cRPMI medium was changed on the day of infection (day 8), and mature macrophages were treated with fresh cRPMI medium. *C. parapsilosis* isolates grown overnight were washed thrice with PBS, and the desired inoculum was used to infect the macrophages. The macrophages were infected at a multiplicity of infection (MOI) of 5 (2.5 million yeast/500,000 macrophages) and incubated for one hour in a CO_2_ incubator at 37°C. Subsequently, the macrophages were extensively washed with PBS, lysed with cold water, and the lysates were plated on YPD agar and incubated at 37°C for 48 hours. Notably, the initial inoculum of each isolate was plated onto YPD agar plates at 37°C for 48 hours for normalization purposes. Phagocytosis was calculated by the number of CFUs of the macrophage lysate relative to the initial inoculum of each isolate, and the values are presented as percentages.

### Primary human neutrophil isolation and infection

Primary human neutrophils were obtained from the blood samples of at least two healthy donors following an approved IRB protocol (#2014P002377). Primary human neutrophils were isolated using the EasySep™ Human Neutrophil Isolation Kit (STEMCELL Technologies), and purity and viability were assessed according to the manufacturer’s instructions. A previously published neutrophil multiparametric assay was utilized [30], which is able to determine the most prominent neutrophil effector functions, i.e. reactive oxygen species (ROS) production, degranulation (CD66b) staining, and phagocytosis. Briefly, neutrophils were infected with FITC-stained *C. parapsilosis* isolates at an MOI of 3, one hour post-infection. Measurements included phagocytosis, ROS production (dihydrorhodamine 123, also known as DHR123), and degranulation using a Celesta BD FACS Flow cytometer (BD Corporation). Following one hour of incubation, the neutrophils were placed on ice for 10 minutes, centrifuged at 4°C and 550 g for 5 minutes, treated with 50 µL FACS buffer (1% heat-inactivated FBS and 3 mM of EDTA) containing anti-human CD66b-antibody (:/300 dilution), incubated on ice in dark for 30 minutes, followed by adding 150 µL extra FACS buffer and centrifugation at 4°C and 550 g for 5 minutes. The neutrophil-*C. parapsilosis* cocultures were resuspended in 200 µL of FACS buffer and subjected to flow cytometry. The gating strategy was set as described previously and 10,000 events were collected per sample. To determine the neutrophil fungicidal activity and survival rate of *C. parapsilosis* isolates following phagocytosis, neutrophils were infected with unlabelled *C. parapsilosis* isolates at an MOI of 0.3 (1 yeast/3 neutrophils), incubated in a CO_2_ incubator at 37°C for 2 hours, and lysed with PBS containing 0.02% Triton X (Millipore Sigma). Lysed neutrophils were plated onto YPD agar plates and incubated at 37°C for 48 hours. Similarly, the initial inoculum of each isolate was plated on YPD agar, and CFUs were determined after 48 hours of incubation at 37°C. *C. parapsilosis* survival rate was assessed by normalizing the CFU of lysed neutrophils to the initial inoculum of each isolate, and the viability values are presented as percentages.

### Cell wall analysis

Cell wall analysis for mannan, beta-glucan, and chitin cell wall constituents was performed using a previously reported flow cytometry-based protocol [29]. Briefly, overnight-grown *C. parapsilosis* isolates in YPD were washed thrice with PBS and subjected to staining with wheat germ agglutinin (WGA)-FITC (ThermoFisher Scientific), AlexaFluor 647 ConA (ThermoFisher Scientific), and an anti-beta-glucan antibody (Biosupplies) to measure chitin, mannan, and beta-glucan, respectively. The stained *C. parapsilosis* isolates were subjected to flow cytometry, and 10,000 events were recorded for each sample. The mean fluorescent intensity (MFI) was recorded for each replicate.

### *In vivo* mouse systemic infection model

Six-week-old female C57BL/6 immunocompetent mice were used, and bloodstream infection was performed in the IACUC-accredited mouse facility at Massachusetts General Hospital following an approved IACUC protocol (#2017N000058). On the day of infection, the overnight-grown *C. parapsilosis* isolates were washed thrice with PBS, and the mice were injected with 200 µL of 10^7^ CFU of *C. parapsilosis* isolates via the tail vein. Six mice per isolate were infected and three mice per isolate group were sacrificed at the designated timepoints (3- and 6-days post-infection). Mice were humanely euthanized prior to sacrifice, and both kidneys, the spleen, and the liver were collected and homogenized. After extensive homogenization, homogenate was plated onto YPD agar plates containing 0.5% pen-strep to prevent bacterial contamination and incubated in a 37°C incubator for 48–72 hours. The organ fungal burden values are presented as CFU/g for each organ.

### Results and discussion

ECR and MDR *C. parapsilosis* are emerging infectious disease challenges. Our multidimensional analysis showed that isolates featuring these phenotypes do not suffer attenuated virulence, as shown by comparable fungal burdens in various organs *in vivo* and attenuated biofilm formation relative to susceptible counterparts obtained from the same hospital. The WGS data also highlights the emergence of another MDR genotype closely related to the previous one. Using a combination of multidimensional *ex vivo*, *in vivo,* and epidemiological approaches, this study suggests that ECR and the majority of the MDR *C. parapsilosis* isolates can cause future outbreaks.

**Figure 1.**
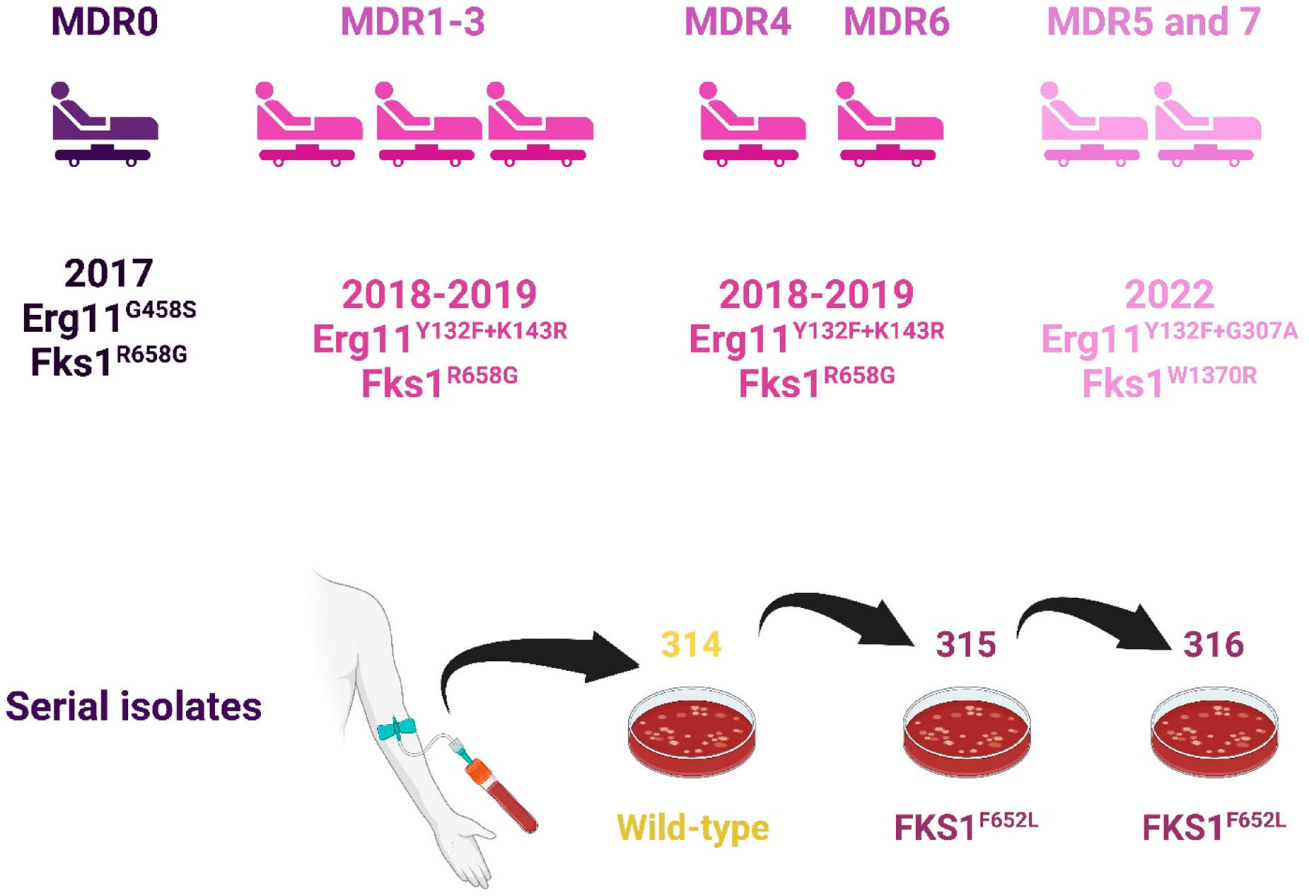
Patients schematic diagram and the *C. parapsilosis* isolates collected from them.

**Figure 2.**
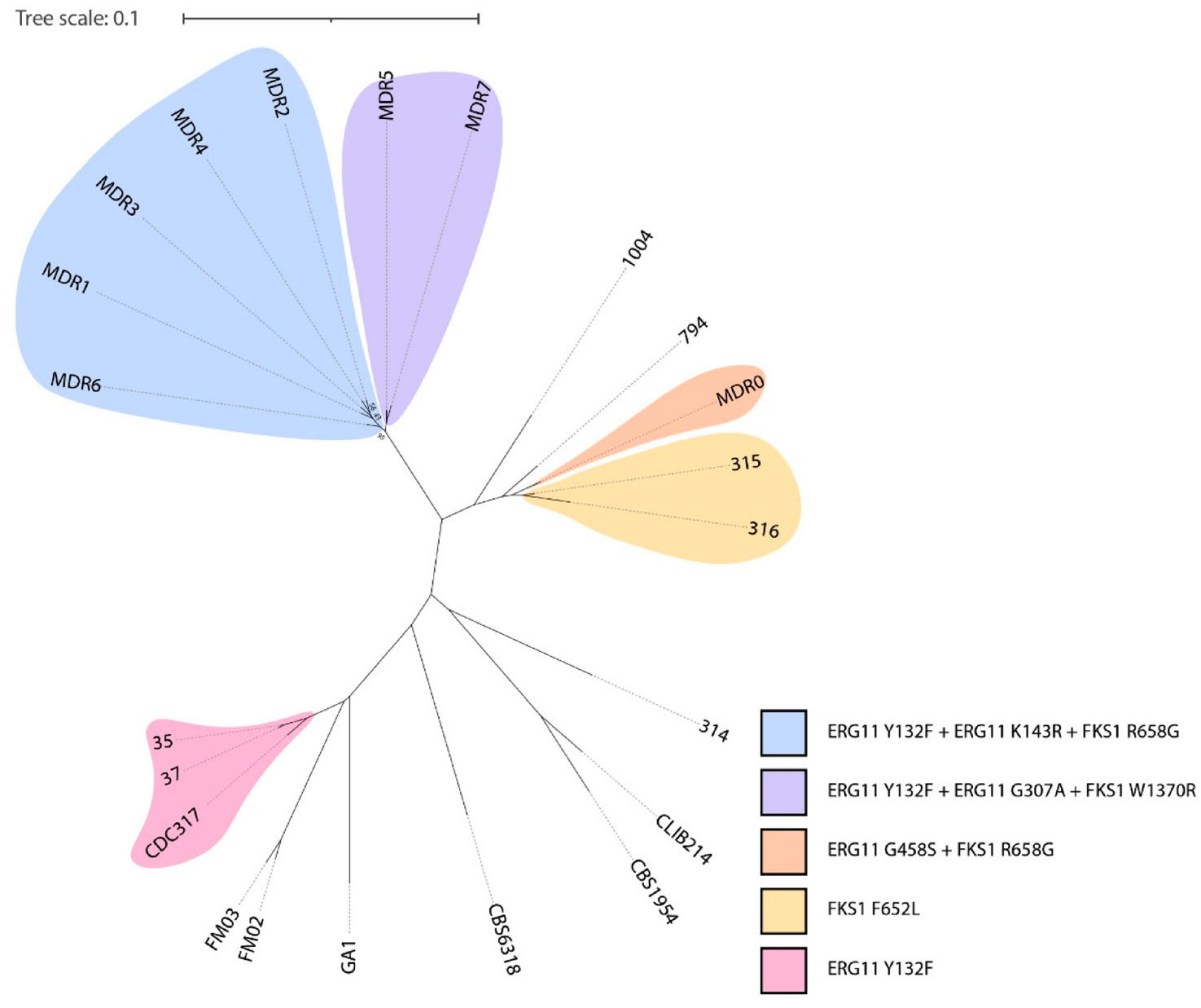
Whole-genome sequence analysis of *C. parapsilosis* isolates. SNP-based phylogeny of *C. parapsilosis* isolates. Variants in Erg11 and Fks1 are shown; the colours represent the patterns of variants present. Bootstraps below 100% are shown.

To assess the genetic relatedness of the newly isolated MDR *C. parapsilosis* isolates (MDR5-7) (Figure 1), WGS and a comparative analysis were performed. Multiple correspondence analysis revealed that three new MDR isolates (MDR5, 6, and 7) group with the MDR1-4 cluster described previously [8,9] (Supplementary Figure 1). These relationships were supported and further characterized by phylogenetic analysis (Figure 2 and Supplementary Figure 1). *C. parapsilosis* MDR1-7 clustered together with a bootstrap value of 100%. There are two sublineages, the primary lineage (MDR1-4, MDR6) and a secondary lineage (MDR5 and 7), that separate from the primary lineage with a bootstrap value of 95%. The MDR1-4 isolates were collected between 2018 and 2020 and were originally described by Daneshnia *et al.*, and MDR6 (collected in 2022) is described here for the first time (Table 1). All the isolates in the primary sublineage (MDR1-4 and MDR6) contained Y132F and a K143R substitution in Erg11, which is also associated with azole resistance [31] (Figure 2 and Supplementary Figure 1). MDR5 and MDR7 in the secondary lineage also contain Y132F, but instead of K143R, they have a different substitution in Erg11 (G307A), which has also been associated with azole resistance [32]. All isolates (MDR1-7) are resistant to both azoles and echinocandins (Table 1). In the primary lineage, ECR is associated with an R658G variant in Fks1 [9,11]. In the secondary lineage, ECR is most likely caused by a different variant, W1370R, in Fks1, which has been identified in ECR isolates of *C. parapsilosis* evolved following exposure to echinocandins [13]. The two sublineages are therefore distinguishable, even though they are closely related and likely share a recent common ancestor. This suggests that clonal outbreaks are associated with closely related isolates. All patients infected with MDR1-7 isolates were hospitalized in the pediatric surgery ward, which was speculated to be the location of the outbreaks. Unfortunately, one of the patients who did not receive the liposomal amphotericin B (LAmB) formulation died, whereas the other two survived upon completion of the LAmB treatment. Therefore, patients infected with MDR strains only respond to LAmB, and treatment with other antifungal drugs was associated with poor prognosis, which underscores the need for prompt identification of the MDR phenotype along with proper antifungal treatment.

The *C. parapsilosis* isolate MDR0 was originally described as echinocandin resistant and is associated with the R658G variant in Fks1 [9]. Here, we noticed that MDR0 also had a heterozygous G458S variant in Erg11 (Figure 2 and Supplementary Figure 1). This variant has been shown to cause azole resistance in *Candida orthopsilosis* [33] and therefore it is an MDR isolate. Interestingly, our WGS analysis revealed an amplification of *ERG11* and the adjacent gene *CPAR2*_303750 in MDR0, increasing the copy number to 15 (Supplementary Table 2, also noted by [32]). Analysis of paired reads indicates that the amplification is in tandem. Some pairs map to opposite ends of the amplified region in inverse orientation, suggesting that the amplifications are in tandem. This increase in the *ERG11* copy number is consistent with our previous studies, wherein we showed that FLCR *C. parapsilosis* isolates carrying the same mutation had a significantly higher basal *ERG11* expression [34]. All other isolates studied had 2–3 copies of *ERG11* (Supplementary Table 2). Amplification of the *ERG11* copy number and its association with azole resistance in *C. parapsilosis* has also been noted in recent studies [32,35]. Copy Number Variations (CNVs) are thought to drive azole tolerance in *C. albicans*, as these genomic events are fairly unstable and disappear upon growth in media lacking the selective pressure azoles [36]. Therefore, such plastic genomic changes may serve as an intermediate step before stable genetic mutations in drug targets are acquired, leading to azole resistance [1]. It would be interesting to explore the impact of *ERG11* copy number variation on azole MIC values and fitness. Alternatively, Erg^G458S^ may have a lower catalytic activity than the WT allele, and this greater copy number may reflect a compensatory mechanism for sufficient ergosterol production as this CNV level has been only noted among the FLCR *C. parapsilosis* isolates recovered from the Ege University Hospital [34].

*C. parapsilosis* isolates 314, 315, and 316 all originated from the same patient. Isolate 315 and 316 are resistant to echinocandins, whereas isolate 314 is susceptible. Isolate 314 is unrelated to isolates 315 and 316, which were more closely related to MDR0, suggesting that the patient had been infected with multiple *C. parapsilosis* genotypes. Isolates 315 and 316 harbour an F652L variant in Fks1. A mutation in the same position, albeit resulting in a different amino acid (F652S), was previously implicated in echinocandin resistance [7]. Interestingly, despite the fact that isolate 314 was initially isolated from a blood sample, isolates 315 and 316 dominated later blood samples even prior to commencement of echinocandin treatment. This potentially indicates that the ECR isolates were fitter and outcompeted isolate 314. Additionally, the ECR isolates may have an environmental origin given that they carried Fks1 mutations and phenotypically were ECR even before echinocandin treatment.

**Figure 3.**
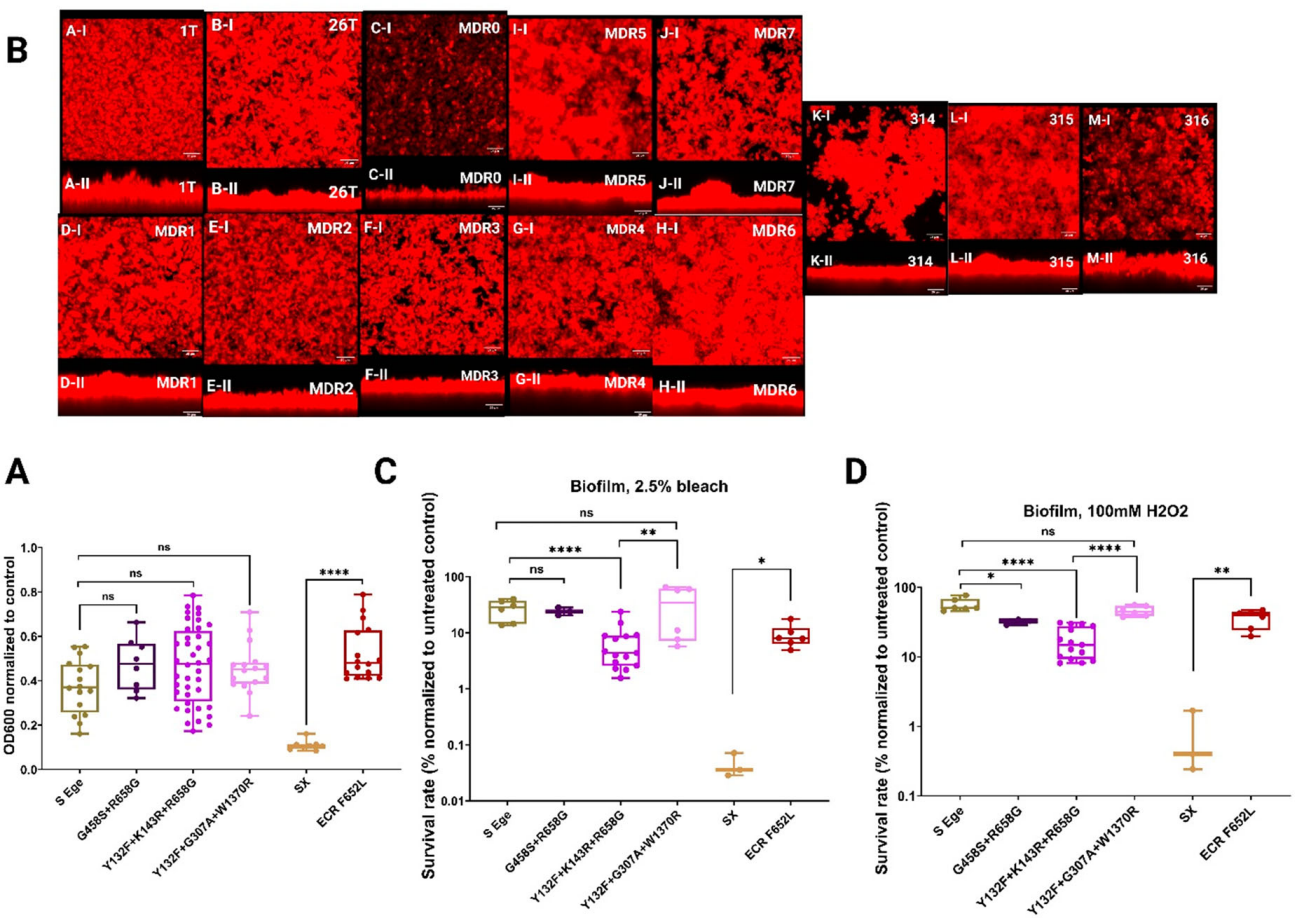
Assessment of the biofilms produced by *C. parapsilosis* isolates. Biofilm optical density assay on polystyrene plates (A). Confocal scanning laser microscopy biofilm assay on silicone squares displaying top-views (upper panels) and side-views (lower panels) of mature biofilms (B); scale bars = 25 μm. Biofilm tolerance to 2.5% bleach (C) and 100 mM of hydrogen peroxide (D). Two-tailed T-tests were used to assess statistical significance and values ≤0.05, ≤ 0.01, and ≤0.001 are shown as *, **, and ****, respectively. S-Ege refers to susceptible isolates from EUH (1 T and 26 T), whereas Sx refers to susceptible isolate #314. G458S + R658G represents MDR0, Y132F + K143R + R658G features MDR1-4 and 6, Y132F + G307A + W1370R refers to MDR5 and 7, whereas ECR F652L represents the ECR isolates #315 and #316.

Given that *C. parapsilosis* outbreaks are thought to be mainly due to the isolate’s abilities to form biofilms, which allows resilience in the environment and host, the biofilm formation capacity and tolerance of *C. parapsilosis* biofilms to compounds widely used in disinfectants (hydrogen peroxide and sodium hypochlorite (bleach) was assessed. In general, we found that the MDR and ECR *C. parapsilosis* isolates produced similar levels of biofilm relative to the susceptible isolates using a standard optical density biofilm assay (Figure 3A). It is noteworthy that the ECR isolates had significantly higher biofilm levels than the susceptible isolate 314 (SX) (Figure 3A). Diving into our WGS data identified 566 homozygous variants from isolate #314. Of particular interest was a nonsynonymous mutation in *TRY5* which has been shown to be involved in biofilm formation, whose deletion resulted in biofilm reduction [37]. Understanding the role of this mutation in biofilm formation requires further functional studies in the future. To further investigate the biofilms formed by these isolates, we observed the morphology of their mature biofilms by confocal scanning laser microscopy. Interestingly, MDR0 had the thinnest and least dense biofilm compared to the other MDR isolates and susceptible isolates from the EUH, whereas the susceptible isolate (#314) had dramatically thinner and less dense mature biofilms compared to the ECR isolates (#315 and 316) (Figure 3B). Additionally, the tolerance of the mature biofilms to 100 mM H_2_O_2_ and 2.5% bleach was tested, which are commonly used disinfectants. Interestingly, the biofilms produced by the historic MDR isolates (MDR1-4 and MDR6) had significantly lower tolerances than those produced by the novel MDR genotypes (MDR5 and MDR7), MDR0, and the susceptible isolates (Figure 3C and 3D). Keeping in mind that the serial isolates belonged to different genotypes, the biofilms of the ECR isolates had significantly greater tolerance to those compounds than did isolate #314 (Figure 3C). Taken together, these findings indicated that the biofilm capacities and biofilm tolerances of the MDR and ECR isolates to H_2_O_2_ and bleach were comparable to susceptible isolates, which could explain why they may resiliently contaminate the hospital environment with a strength comparable to that of their susceptible counterparts. Similarly, given that robust biofilm formation requires effective environmental colonization, the rare occurrence of MDR0 and the susceptible isolate (#314) could be due to a decreased biofilm level.

**Figure 4.**
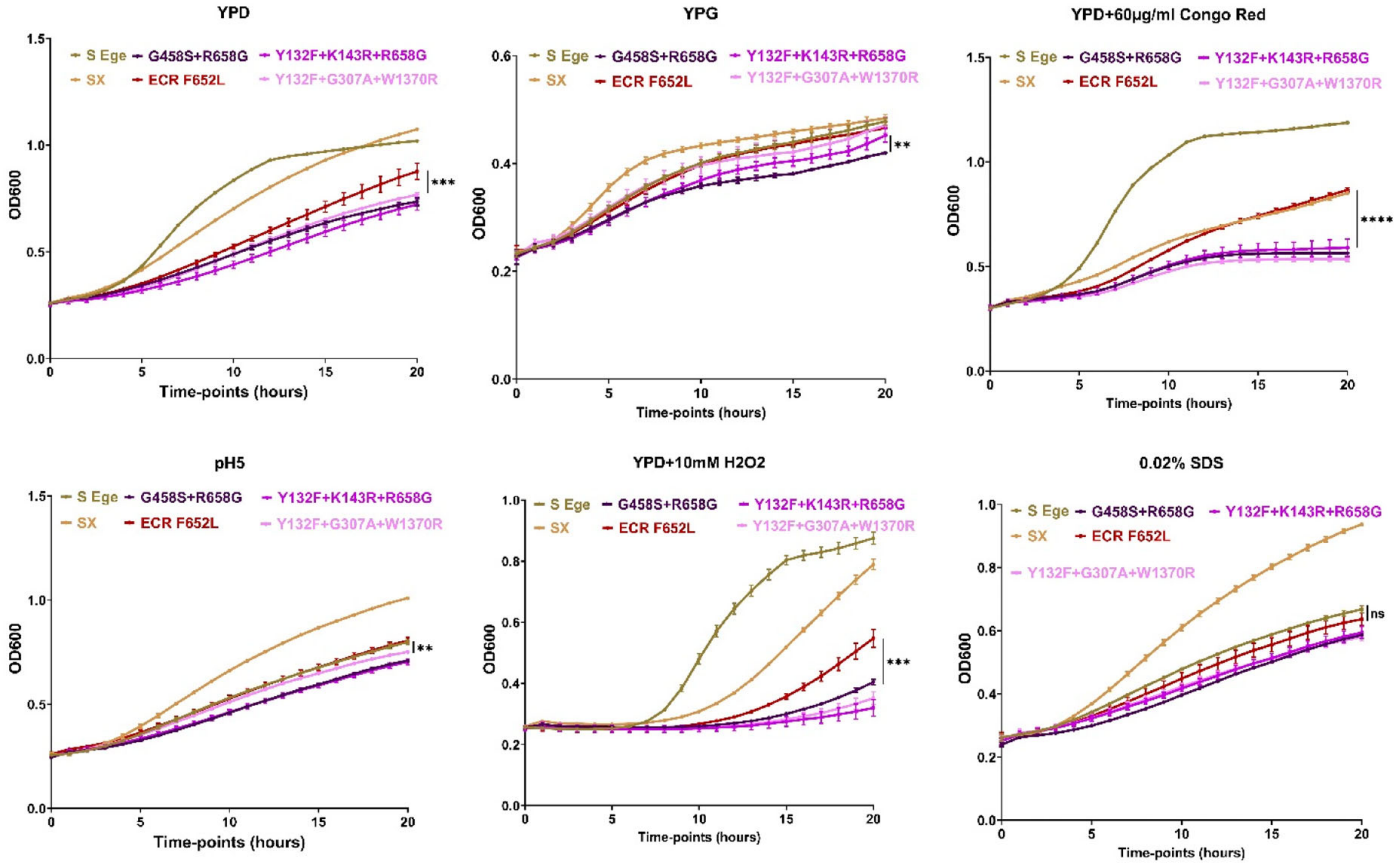
*In vitro* growth assessment in laboratory media with/without stressors. Two-tailed T-tests were used to assess statistical significance and values ≤0.05, ≤ 0.01, and ≤0.001 are shown as *, **, and ****, respectively. S-Ege refers to susceptible isolates from EUH (1 T and 26 T), whereas Sx refers to susceptible isolate #314. G458S + R658G represents MDR0, Y132F + K143R + R658G features MDR1-4 and 6, Y132F + G307A + W1370R refers to MDR5 and 7, whereas ECR F652L represents the ECR isolates #315 and #316.

Next, we investigated the fitness of the same isolates in the context of the host conditions. We began with an *in vitro* growth assay, which included growth rate determination in YPD in the presence and absence of a stressor over a 20-hour timepoint. In general, the susceptible isolates had a significantly greater growth rate than the susceptible isolates, followed by the ECR and MDR isolates (except for at pH 5 and in the presence of SDS). Interestingly, the novel MDR isolate had a slightly greater growth rate than the historic ones (MDR1-4 and 6) and the single MDR isolate (MDR0) had a significantly lower growth rate in laboratory media supplemented with an alternative carbon source, glycerol (Figure 4). Therefore, compared with susceptible isolates, ECR and MDR isolates exhibit some degree of fitness cost under certain conditions. These growth defects have also been noted in ECR *C. parapsilosis* isolates that evolved *in vitro* [13], but not in ECR mutants carrying R658G generated by CRISPR-Cas9 from ATCC 22019 [9]. Nonetheless, the higher fitness cost of the MDR isolates could be because they harbour mutations in both Erg11 as well as the Fks1.

**Figure 5.**
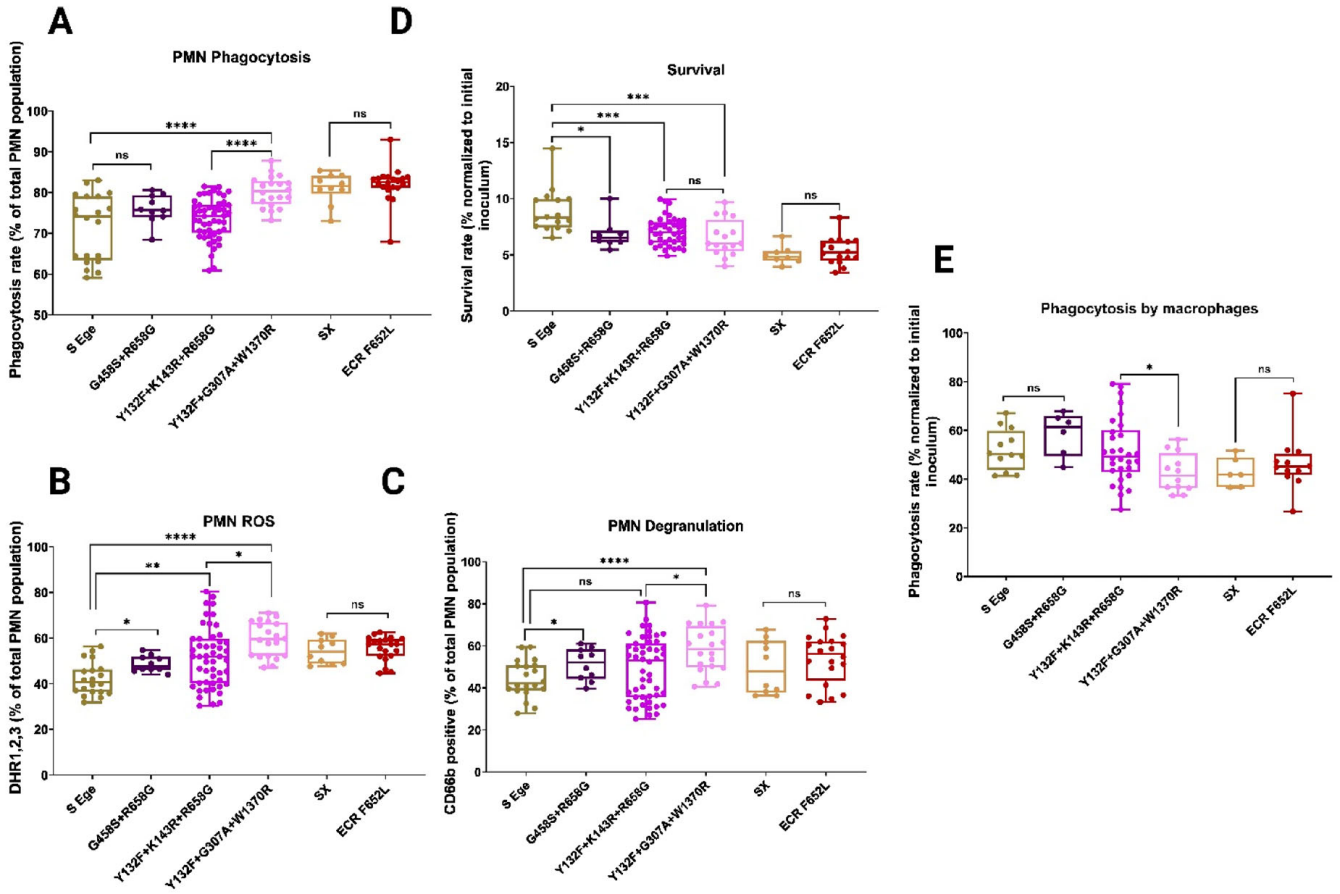
Interaction of *C. parapsilosis* isolates with primary human neutrophils and macrophages. Neutrophil phagocytosis (A), reactive oxygen species production (B), degranulation (C), and survival of *C. parapsilosis* isolates after two hours incubation with neutrophils (D). Macrophage phagocytosis (E). Two-tailed T-tests were used to assess statistical significance and values ≤0.05, ≤ 0.01, and ≤0.001 are shown as *, **, and ****, respectively. S-Ege refers to susceptible isolates from EUH (1 T and 26 T), whereas Sx refers to susceptible isolate #314. G458S + R658G represents MDR0, Y132F + K143R + R658G features MDR1-4 and 6, Y132F + G307A + W1370R refers to MDR5 and 7, whereas ECR F652L represents the ECR isolates #315 and #316.

Subsequently, we assessed the interaction and fitness of these isolates while coculturing them with primary human neutrophils and macrophages. Interestingly, the novel MDR isolates (MDR5 and 7) were significantly more engulfed by neutrophils compared to other isolates (Figure 5A), resulting in higher ROS levels and degranulation by neutrophils (Figure 5B and 5C); therefore, these novel MDR isolates did not survive as well as other isolates (Figure 5D). Notably, the lower survival of the novel MDR isolates compared to the susceptible isolates is not necessarily an indication of lower fitness since the neutrophil phagosomes have extremely high fungicidal activity, and the less neutrophil engulfment there is, the greater the viability of the *C. parapsilosis* isolates. Similarly, despite being significantly more engulfed by neutrophils, the novel MDR isolates had very similar viability compared to the historic MDR isolates (MDR1-4 and 6), which may indicate that the novel MDR isolates are potentially more tolerant to hostile neutrophil phagosome environments or that they may interfere with phagolysosome maturation. The serial isolates were engulfed at the same rate, triggered similar ROS and degranulation, and had similar viabilities (Figure 5A-D). Next, we assessed the interaction of these *C. parapsilosis* isolates with primary human macrophages. Since macrophages exhibit fungistatic activity, unlike the fungicidal activity of neutrophils, and because *C. parapsilosis* pseudohyphae lyse macrophages at later timepoints, our *C. parapsilosis*-macrophage analysis focused only on phagocytosis. Intriguingly, in contrast to the neutrophil data, the novel MDR isolates and the serial isolates (314-316) were significantly less strongly phagocytosed by primary human macrophages, in contrast to the neutrophil phagocytosis trend (Figure 5E). In general, interactions with primary human neutrophils and macrophages indicated that the novel MDR and ECR isolates had comparable fitness levels, as measured by neutrophil viability and macrophage evasion.

**Figure 6.**
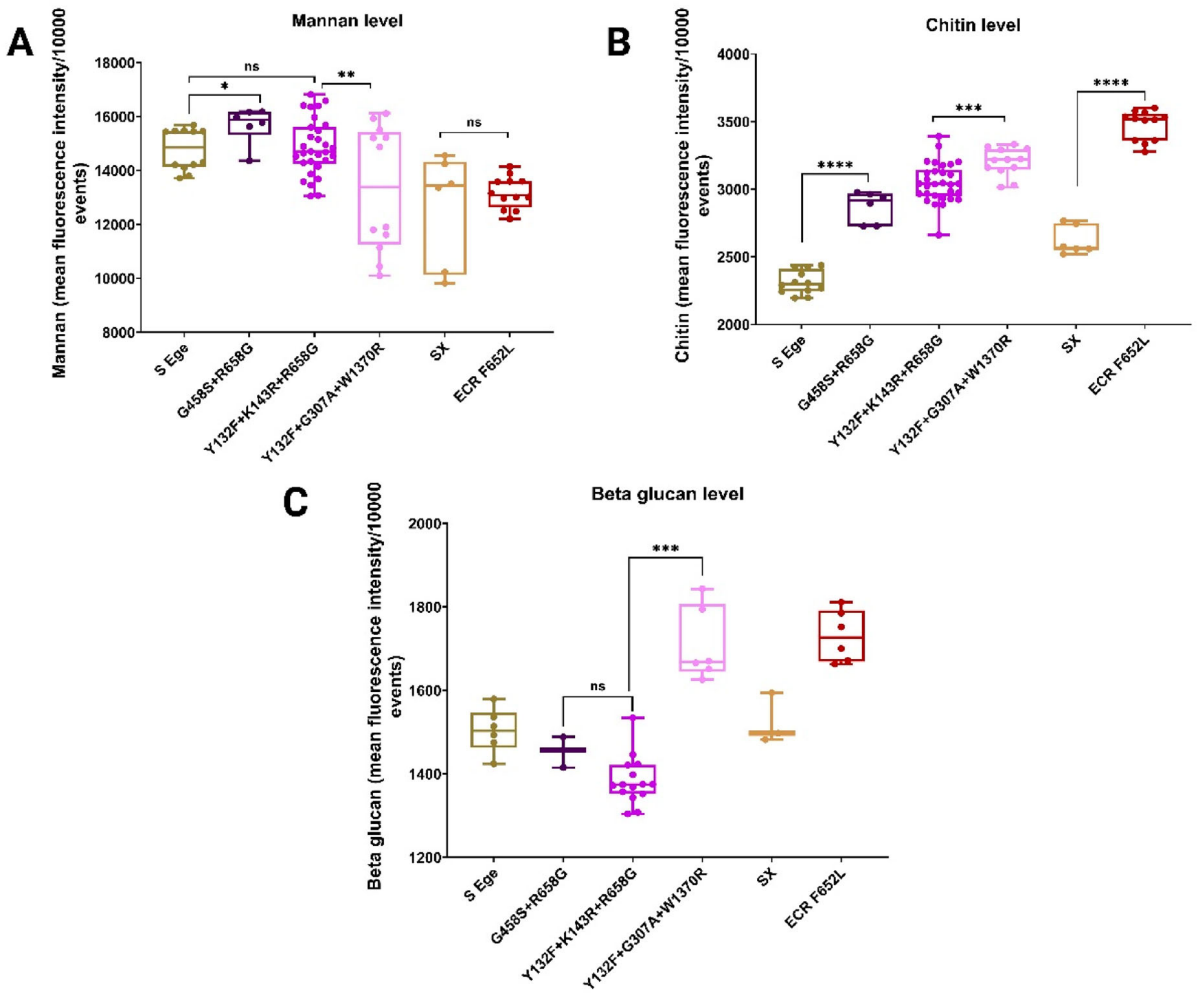
Cell wall analysis and interaction with primary human neutrophils. Flow cytometric analysis of mannan (A), chitin (B), and β-glucan (C) of *C. parapsilosis* isolates. Two-tailed T-tests were used to assess statistical significance and values ≤0.05, ≤ 0.01, and ≤0.001 are shown as *, **, and ****, respectively. S-Ege refers to susceptible isolates from EUH (1 T and 26 T), whereas Sx refers to susceptible isolate #314. G458S + R658G represents MDR0, Y132F + K143R + R658G features MDR1-4 and 6, Y132F + G307A + W1370R refers to MDR5 and 7, whereas ECR F652L represents the ECR isolates #315 and #316.

Given that echinocandin resistance is accompanied by cell wall alterations and that MDR, ECR, and susceptible isolates are differentially phagocytosed by innate immune cells, we performed cell wall analysis of mannan (Figure 6A), chitin (Figure 6B), and β-glucan (Figure 6C). Interestingly, the novel MDR isolates had significantly lower mannan concentrations than the other isolates (Figure 6A). On the other hand, the MDR and ECR isolates had significantly greater exposed chitin levels than their susceptible counterparts (Figure 6B); more specifically, the novel MDR strains had the highest chitin levels, followed by the historical MDR isolates and MDR0. Interestingly, we noted that, compared with the other MDR strains and the pertinent susceptible strain from the same hospital, only the novel MDR isolates (MDR5 and 7) and ECR isolates (315 and 316) had significantly greater exposure levels of β-glucan, whereas the historical MDR isolates had slightly less β-glucan exposure (Figure 6C). In keeping with a previous report [13], these novel MDR isolates exhibited very similar patterns of cell wall changes when compared to *in vitro*-evolved *C. parapsilosis* isolates harbouring W1370R. Since the β-glucan exposure level is known to be a potent immunogenic pathogen-associated molecular pattern recognizable by dectin-1 and because the novel MDR isolates had greater β-glucan exposure than the other MDR and susceptible counterparts from the EUH, we assumed that their greater phagocytosis by neutrophils was driven by dectin-1 recognition, which requires further investigation in our future studies. Taken together, the cell wall analysis indicated that Fks1 amino acid substitutions may differentially impact the cell wall architecture, which in turn dictate interactions with host immune cells and pathogen fate.

**Figure 7.**
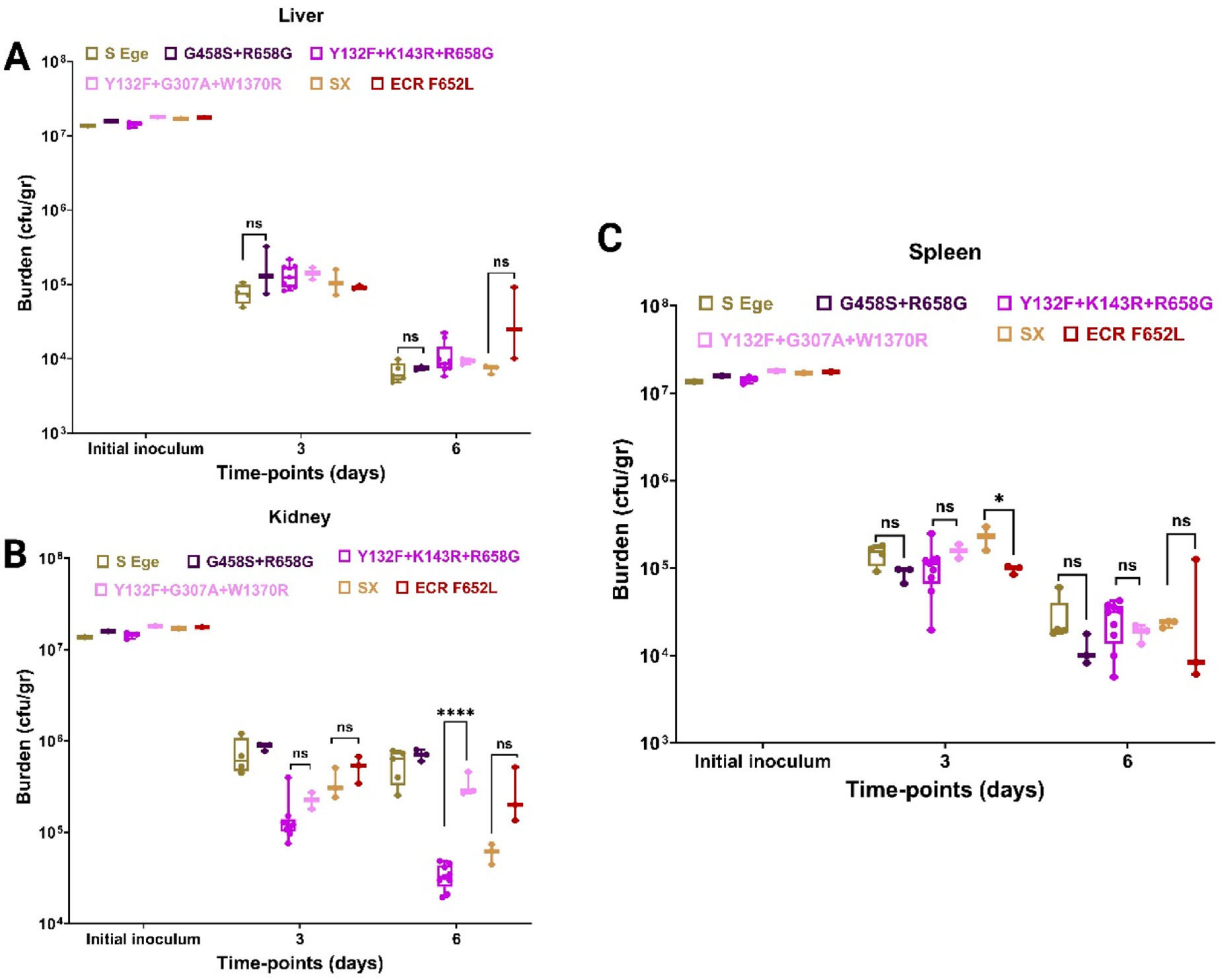
*In vivo* assessment of *C. parapsilosis* isolates (8 isolates, see Table 1 for more details). Immunocompetent mice were infected with 10^7^ colony forming units and fungal burdens in the liver (A), kidneys (B), and spleen (C) were determined on days 3 and 6 post-infection. Two-tailed T-tests were used to assess statistical significance and values ≤0.05, ≤ 0.01, and ≤0.001 are shown as *, **, and ****, respectively. S-Ege refers to susceptible isolate #26 from EUH, whereas Sx refers to susceptible isolate #314. G458S + R658G represents MDR0, Y132F + K143R + R658G features MDR1-4 and 6, Y132F + G307A + W1370R refers to MDR5 and 7, whereas ECR F652L represents the ECR isolates #315 and #316.

To simulate the host and real-life conditions, we induced systemic mouse model infection and assessed the fungal burden in various organs, i.e. the kidneys, liver, and spleen, on days 3 and 6 post-infection. We included 6 isolates from the EUH, including a susceptible strain exhibiting the highest *in vitro* and *ex vivo* fitness (#26); MDR0, one of the novel MDR isolates; and three of the historic MDR isolates collected from different years. Regarding the isolates from the same patient, we included isolates 314 and 316 (see Table 1 for additional details). Interestingly, the novel MDR isolates and the historic MDR isolates had slightly higher fungal burden than their susceptible counterparts in the liver on day six (albeit the differences were not significant), whereas the ECR isolate had a significantly greater fungal burden than its susceptible counterpart (Figure 7A). In the kidneys, the historic MDR isolates had a significantly lower survival than the other EUH isolates (including susceptible and other MDR isolates), whereas the ECR isolate (316) had a significantly greater fungal burden than its susceptible counterpart (314) (Figure 7B). Finally, the novel MDR and the historic MDR isolates had significantly greater fungal burden than MDR0 on day six, whereas there was no significant difference between the serially recovered susceptible and ECR isolates on the same day (Figure 7C). When combined with phagocytosis of the historic MDR strains and their similar spleen and liver fungal burdens (macrophage-rich organs), we hypothesize that the historic MDR isolates may better counteract macrophage responses, whereas the novel MDR isolates may better tolerate neutrophil fungicidal effector functions. Taken together, our *in vivo* animal findings suggest that the MDR isolates generally do not suffer obvious fitness costs in most of the organs tested and that the ECR isolates have a greater fungal burden *in vivo* (compared to 314), which is contrary to the significant fitness costs of the ECR isolates obtained *in vitro* by exposure to echinocandins [13]. This contradiction potentially shows that the evolution of resistance in the context of the host, especially during an outbreak, may result in compensatory mutations suppressing the detrimental effects of drug target mutations. Importantly, our observations also suggest that assessing fitness merely through *in vitro* experiments or using drug-resistant isolates evolved *in vitro* in the absence of host mimicking conditions may provide misleading data, which may underestimate the potential of drug-resistant *C. parapsilosis* isolates to cause persistent infections and/or outbreaks in real life.

In conclusion, our study suggests that the use of epidemiological data over several years along with *in vitro* biofilm and *ex vivo* and *in vivo* data could inform on the resilience of *C. parapsilosis* isolates in hospital settings as well as inform on their potential for causing outbreaks. Accordingly, we anticipate that both the novel MDR and the historic strains have the potential to cause problematic outbreaks in the years to come. Although epidemiological data for ECR isolates are lacking, robust biofilm formation and high tolerance to disinfectants, as well as high organ fungal burdens *in vivo*, may underscore the fact that ECR isolates could lead to serious drug-resistant future outbreaks.

## Supplementary Material

Supplementary_Tables

Supplementary_Figures

## Geolocation information

Türkiye.
