# Supplementary material for: Evaluation of outbreak persistence caused by multidrug-resistant and echinocandin-resistant Candida parapsilosis using multidimensional experimental and epidemiological approaches
Source: Emerg Microbes Infect. 2024 Feb 21;13(1):2322655. doi: 10.1080/22221751.2024.2322655 (PMC10916928; doi:10.1080/22221751.2024.2322655)
Supplement: Supplementary_Tables [file TEMI_A_2322655_SM0644.docx]

**Supplementary Table 1. List of whole-genome sequenced *Candida parapsilosis* isolates used in this study.**

| **Isolate** | **BioProject** | **Citation** |
| --- | --- | --- |
| MDR5 | TBD | This study |
| MDR6 | TBD | This study |
| MDR7 | TBD | This study |
| 314 | TBD | This study |
| 315 | TBD | This study |
| 316 | TBD | This study |
| MDR0 | PRJNA921967 | Daneshnia *et al*., 2023 |
| MDR1 | PRJNA921967 | Daneshnia *et al*., 2023 |
| MDR2 | PRJNA921967 | Daneshnia *et al*., 2023 |
| MDR3 | PRJNA921967 | Daneshnia *et al*., 2023 |
| MDR4 | PRJNA921967 | Daneshnia *et al*., 2023 |
| 35 | PRJNA784379 | Arastehfar *et al*., 2022 |
| 37 | PRJNA784379 | Arastehfar *et al*., 2022 |
| CDC317 | PRJNA795920 | Bergin *et al*., 2022 |
| FM02 | PRJNA795920 | Bergin *et al*., 2022 |
| FM03 | PRJNA795920 | Bergin *et al*., 2022 |
| 795 | PRJNA795920 | Bergin *et al*., 2022 |
| 1004 | PRJNA748054 | Bergin *et al*., 2022 |
| CLIB214 | PRJNA563885 | Ola *et al*., 2020 |
| CBS6318 | PRJEB1685 | Pryszcz *et al*., 2013 |
| CBS1954 | PRJEB1685 | Pryszcz *et al*., 2013 |
| GA1 | PRJEB1685 | Pryszcz *et al*., 2013 |

**Supplementary Table 2 CNVs in *ERG11* and adjacent gene CPAR2_303750.**

| **Isolate** | **ERG11** | **CPAR2_303750** |
| --- | --- | --- |
| 35 | 2 | 2 |
| 37 | 2 | 2 |
| MDR5 | 2 | 2 |
| MDR6 | 2 | 2 |
| MDR7 | 2 | 2 |
| 314 | 2 | 2 |
| 315 | 3 | 3 |
| 316 | 3 | 3 |
| 794 | 3 | 3 |
| 1004 | 2 | 2 |
| CBS1954 | 2 | 2 |
| CBS6318 | 2 | 2 |
| CDC317 | 2 | 2 |
| CLIB214 | 2 | 2 |
| MDR0 | 15 | 15 |
| FM02 | 2 | 2 |
| FM03 | 2 | 2 |
| GA1 | 2 | 2 |
| MDR1 | 2 | 2 |
| MDR2 | 2 | 2 |
| MDR3 | 2 | 2 |
| MDR4 | 2 | 2 |
