# Supplementary figures and images for: Evaluation of outbreak persistence caused by multidrug-resistant and echinocandin-resistant Candida parapsilosis using multidimensional experimental and epidemiological approaches
Source: Emerg Microbes Infect. 2024 Feb 21;13(1):2322655. doi: 10.1080/22221751.2024.2322655 (PMC10916928; doi:10.1080/22221751.2024.2322655)

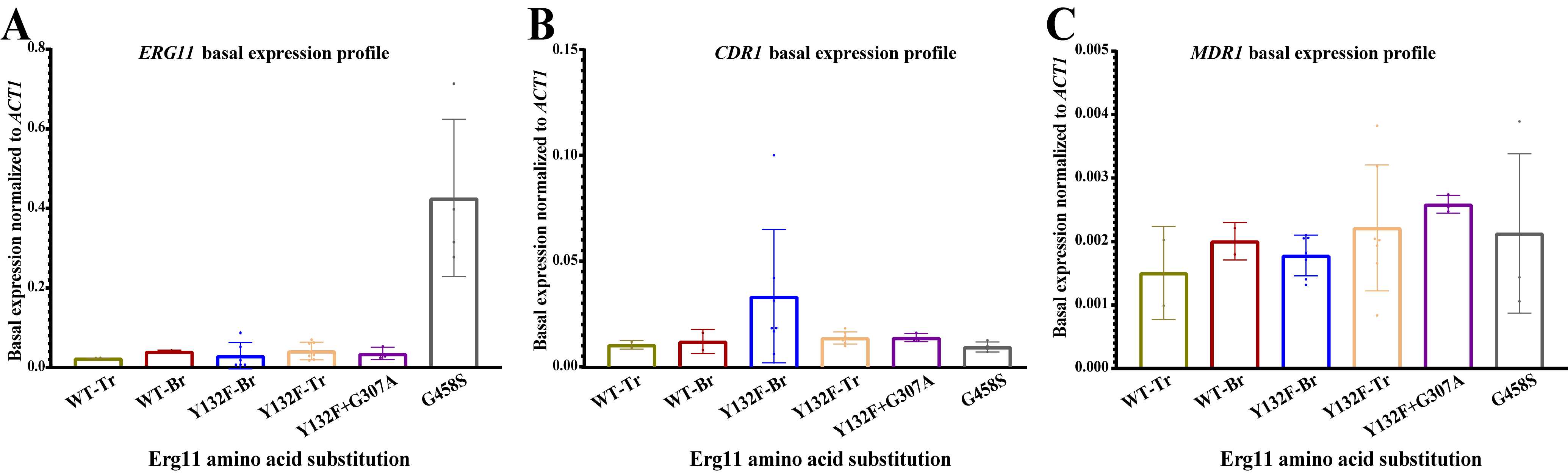

Supplement: Supplementary_Figures [file TEMI_A_2322655_SM0269.zip › Image_1_Determinants of fluconazole resistance and the efficacy of fluconazole and milbemycin oxim combination against Candida parapsilosis clinical i.tif]

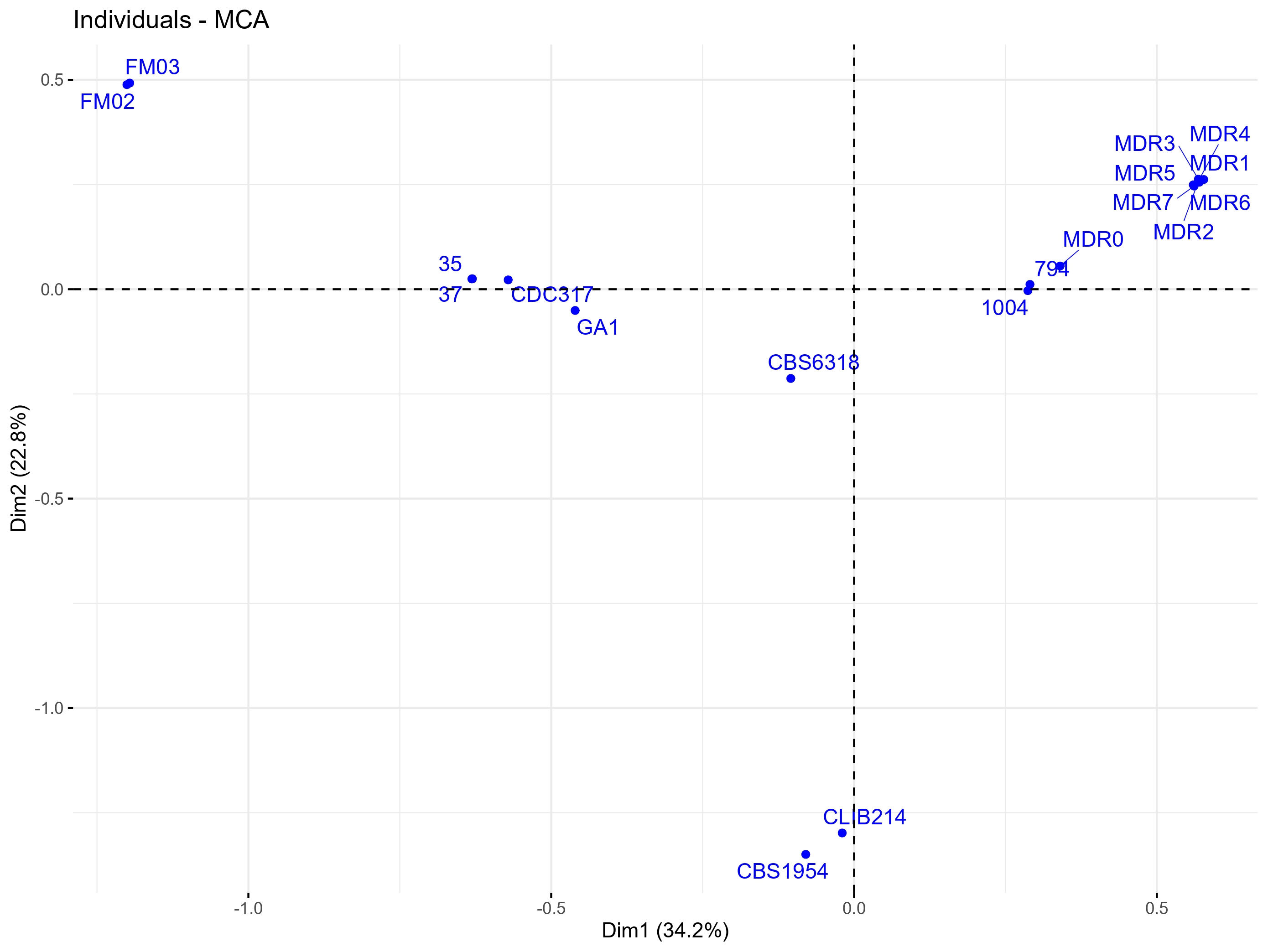

Supplement: Supplementary_Figures [file TEMI_A_2322655_SM0269.zip › Supplementary Figure 1.jpg]
